# A Peculiar Case

**Published:** 1885-07

**Authors:** 


### A Peculiar Case.

In a recent letter from W. A. H. of Michigan, is the following peculiar case: He says, "In the early part of last Autumn I made a superior and inferior set of teeth for a lady, the superior of rubber and the inferior of Watt's Metal. Every thing seemed all right with the case until about January 1st, when a few red and somewhat sore places appeared on the inferior jaw; these soon passed away and the eyes became affected, were very weak and when in a warm room heated by a stove, they would become quite blurred, and she could hardly see. The eye-lids were very much inflamed. This continued for some time, but improved very considerably upon the gums becoming inflamed and sore. This was only of the inferior jaw. Her eyes were treated for some time without success, and finally her physician stated that it was caused from wearing a rubber and metal plate; from this opinion, I dissented and gave her treatment for the gums, which seemed wholly to relieve them, and for some time she has had no return of the disease in that direction, and now her eyes trouble her again. I have never known or heard of such a case, and can not understand how the two plates could affect the eyes in that way, especially when there is no disease now of the gums."

This case is certainly a peculiar one, and though not of common occurrence, it is not very strange. It was perhaps a transfer of irritation by reflex action. Most probably the eyes were originally weak, and some adverse influence present with them, that only required a little excitation to cause acute disease. I am not aware that irritation has been produced in any case in the healthy mouth, simply by the presence of two plates, one of rubber and the other of Watt's Metal, and yet when we consider how many phases of disease are found in the mouth, we are not prepared to say that such an arrangement would not in any case produce irritation. There may have been many cases similar to this that have not been made public. Reflex influences, especially from nervous affections of the mouth reaching out to various parts more or less remote, are of frequent occurrence. These are, however, in many cases very obscure, and oftentimes the most rigid examination produces no very satisfactory result, even in the hands of those who discriminate closely.

